# Spawning stock and recruitment in North Sea cod shaped by food and climate

**DOI:** 10.1098/rspb.2010.1465

**Published:** 2010-09-01

**Authors:** Esben Moland Olsen, Geir Ottersen, Marcos Llope, Kung-Sik Chan, Grégory Beaugrand, Nils Chr. Stenseth

**Affiliations:** 1 Institute of Marine Research Flødevigen, N-4817 His, Norway; 2 Institute of Marine Research, Gaustadallèen 21, 0349 Oslo, Norway; 3 Department of Biology, Centre for Ecological and Evolutionary Synthesis (CEES), University of Oslo, PO Box 1066 Blindern, N-0316 Oslo, Norway; 4 Department of Statistics and Actuarial Science, University of Iowa, Iowa City, IA 52242, USA; 5 Centre National de la Recherche Scientifique, Laboratoire d'Océanologie et de Géosciences', UMR LOG CNRS 8187, Station Marine, Université des Sciences et Technologies de Lille—Lille 1 BP 80, 62930 Wimereux, France

**Keywords:** North Sea cod, climate, modelling, stock–recruitment, zooplankton

## Abstract

In order to provide better fisheries management and conservation decisions, there is a need to discern the underlying relationship between the spawning stock and recruitment of marine fishes, a relationship which is influenced by the environmental conditions. Here, we demonstrate how the environmental conditions (temperature and the food availability for fish larvae) influence the stock–recruitment relationship and indeed what kind of stock–recruitment relationship we might see under different environmental conditions. Using unique zooplankton data from the Continuous Plankton Recorder, we find that food availability (i.e. zooplankton) in essence determines which model applies for the once large North Sea cod (*Gadus morhua*) stock. Further, we show that recruitment is strengthened during cold years and weakened during warm years. Our combined model explained 45 per cent of the total variance in cod recruitment, while the traditional Ricker and Beverton–Holt models only explained about 10 per cent. Specifically, our approach predicts that a full recovery of the North Sea cod stock might not be expected until the environment becomes more favourable.

## 1. Introduction

For both fisheries and conservation purposes, a major challenge is to understand how environmental change influences marine ecosystems [1,2]. For instance, climate change may influence recruitment of marine fishes directly through physiological processes and also indirectly through temperature-induced shifts in the zooplankton community, representing the main food items for newborn fishes [3,4]. Theory suggests that such effects may modify the quantitative relationship between the mature population and the recruits, i.e. the stock–recruitment curves [5]. Unfortunately, discerning the underlying stock–recruitment relationship has remained a central and long-lasting problem in fisheries science [6]. Although there is good evidence showing that high recruitment tends to occur when spawner abundance is high, and vice versa [7], the value of fitting specific stock–recruitment relationships in marine fishes has been questioned owing to the weak associations that are frequently observed [6,8]. A poor fit may be simply owing to insufficient data and/or inadequate models, and a general denial of meaningful stock–recruitment relationships would have rather alarming consequences on the science of fish population dynamics [9]. A more constructive approach would be to expand upon the established knowledge, both by introducing refined models and by including other sources of data [10,11]. The work presented here is intended to be a contribution towards this means.

In their pioneering study, Cushing & Horwood [12] found unexpected support for strong density-dependent mortality of fish larvae, even with low observed larval densities. The model indicates that there is a positive link between the number of food organisms and the slope of the stock–recruitment relationship, with potentially important implications for population dynamics [13]. Chaotic-like dynamics may exist depending on food availability for fish larvae and fishing pressure on the recruited individuals [14]. More recently, a theoretical study by Johansen [5] found that under abundant food availability, the recruitment curve might become monotonically increasing towards an upper limit, i.e. a Beverton–Holt type stock–recruitment relationship [6,15], whereas a Ricker-type stock–recruitment relationship incorporating the feature of overcompensation [6,16] was expected at limited food levels. Specifically, Johansen [5] suggested that lack of food will slow down the growth of the larvae and delay the time to metamorphosis (the end of the larval stage), and that this may cause the larval cohort to experience density-dependent mortality to the extent that the recruitment curve becomes over-compensatory (see also [17]).

Here, we test this expectation by developing a modelling approach linking the traditional Ricker and Beverton–Holt stock–recruitment models so that the Beverton–Holt model will have most weight when food availability for fish larvae is good and the Ricker model will have most weight when food availability is sparse (see §2; electronic supplementary material). We apply this model to long-term data on cod (*Gadus morhua* L.) in the North Sea, where excellent long-term data on zooplankton (i.e. food for fish larvae) are available [18]. The North Sea cod has been in a poor state for several years, partly owing to overfishing and partly owing to environmental change [19,20]. Furthermore, North Sea cod is at the southern edge of this species distribution and plausible scenarios of future climate change are expected to slow the recovery of the stock [21]. Recent empirical studies provide evidence that shifts in the composition of the zooplankton community, linked to shifts in the temperature regime, have had a clear influence on cod recruitment [4,20,22]. Sea temperature may also act directly on recruitment [3,23] and indirectly via the spatial distribution of the spawning stock [24]. The current understanding suggests a particularly strong effect of climate through plankton during the larval stage of cod development [4]. Here, we test for both a plankton effect and a temperature effect on cod recruitment.

We find evidence that both the shape and the position of the underlying stock–recruitment relationship are not fixed. Instead, a family of recruitment functions may result from variability in environmental conditions. This has implications for the management of harvested species such as the North Sea cod because it strongly suggests that recruitment effects of harvesting (i.e. reducing the spawning stock) and climate change will not be independent.

## 2. Material and methods

### (a) The Atlantic cod

The Atlantic cod is an important food fish found along both the western and eastern parts of the North Atlantic Ocean. Many of the historically large populations have been severely depleted by harvesting, including cod in the North Sea basin [19]. The Atlantic cod is a highly fecund bet-hedging species with no parental care [25,26]. Spawning typically occurs during February to May, depending on the location [27], and involves complex behaviour within and between sexes [28]. In the North Sea, the peak of the spawning period is usually in March [29]. The offspring of Atlantic cod spend from several weeks up to five months as pelagic eggs and larvae and then settle towards the bottom as age 0 juveniles [30]. During the pelagic stage, the larvae feed on energy-rich zooplankton such as *Calanus finmarchicus* [31].

### (b) Datasets

**Figure 1.**
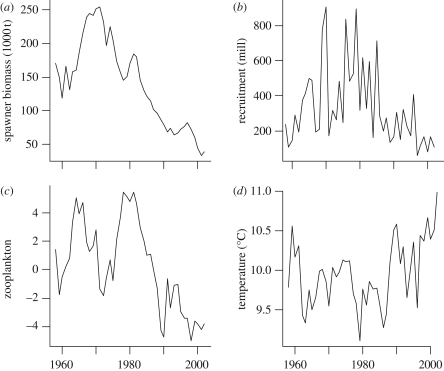
Data series. Temporal variation in (*a*) North Sea cod spawning stock biomass, (*b*) recruitment at age 1 year, (*c*) zooplankton index, and (*d*) sea surface temperature (SST).

We used data series from the available period of overlap 1958–2002 (figure 1; see also electronic supplementary material). Data on North Sea cod spawning stock biomass and recruitment were obtained from www.ices.dk, following the procedure described by Beaugrand *et al*. [20]. These data were derived from virtual population analysis [32]. Recruitment refers to the estimated numbers of fishes at age 1. This is the stage where the fishes first enter the fisheries and the earliest measure of year class strength available for the full time period. Zooplankton data are from the Continuous Plankton Recorder survey, where samples were collected by merchant ships continuously towing a plankton recorder on their regular routes in the North Atlantic and the North Sea [18,33]. A plankton index was developed using the procedure proposed by Beaugrand *et al*. [20]. This index represents the first principal component performed on key zooplankton indicators of food for larval cod [20]. A positive value reflects high total calanoid copepod biomass, large mean size of calanoid copepods, high abundance of abundance of *C. finmarchicus* and euphausiids and low abundance of *Calanus helgolandicus*. The plankton index was scaled to range between zero and one. Sea surface temperatures (SSTs) were obtained from the International Comprehensive Ocean–Atmosphere DataSet (ICOADS, 1-degree enhanced) provided by the NOAA-ESRL Physical Sciences Division, Boulder CO, USA (http://www.cdc.noaa.gov/). Annual averaged values (°C) were calculated for the North Sea–Skagerrak area (from longitude −3.5–11.5° E and latitude 51.5–61.5° N). SST is standardized to a mean of zero and a standard deviation of one.

### (c) Stock–recruitment modelling

We fitted a combined Ricker–Beverton–Holt model incorporating food availability (zooplankton, *Z*) and climate (sea temperature, *T*) to the available stock (*S*) and recruitment (*R*) data on North Sea cod (table 1, model 1). On the original scale, this model can be expressed as:
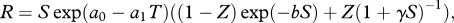

where *γ* = exp(*c*)/max *S*, and max *S* is the maximum observed spawning biomass. The reproductive rate equals exp (*a*_0_ −*a*_1_*T*)((1−*Z*) exp(−*bS*) + *Z*(1 + *γS*)^−1^), with exp (*a*_0_ −*a*_1_*T*) as the maximum reproductive rate at temperature *T*. The rescaling of the spawning biomass by its observed maximum value makes the variables comparable in their scales, which avoid multi-collinearity [34]. For interpretation, it can be absorbed into exp(*c*). The parameterization exp(*c*) ensures that the coefficient *γ* is positive, which prevents numerical problems in fitting the model. While the parameter *b* is also postulated to be positive, it does not create numerical problems in fitting by not constraining it to be positive, and hence no such constraint was imposed. Note that the term ((1 − *Z*) exp(−*bS*) + *Z*(1 + *γS*)^−1^) represents the density dependence, which is now a function of the zooplankton index *Z*. As *Z* tends to 1, the density dependence approaches the form of Ricker model with an exponential decay in the density, namely exp(−*bS*), whereas as *Z* tends to 0, the density dependence is asymptotically Beverton–Holt, namely (1 + *γS*)^−1^. For a fractional *Z*, the density dependence is a weighted mean of these two types of density dependence with weight *Z* for the Beverton–Holt density dependence. In particular, when *Z* = 0, the model becomes the Ricker model but it reduces to the Beverton–Holt model when *Z* = 1.

**Table 1.** Stock (*S*) and recruitment (*R*) models fitted to North Sea cod and environmental data (figure 1): (1) a combined Ricker–Beverton–Holt model including zooplankton (*Z*) and temperature (*T*) effects, (2) a combined Ricker–Beverton–Holt model including a zooplankton effect only, (3) a traditional Ricker model, (4) a traditional Beverton–Holt model, (5) a Ricker model including a zooplankton effect and (6) a Beverton–Holt model including a zooplankton effect. (Model formulation is on the logarithmic scale because this ensures that the constant-variance and normal error distribution assumptions are better met (see electronic supplementary material). (The term max*S* refers to the maximum observed spawning stock biomass (included in order to normalise the spawing stock biomass and avoid problems of colinearity), while *a*_0_, *a*_1_, *b* and *c* are parameters to be estimated.))

model	structure
1	log(*R*) − log(*S*) = *a*_0_*− a*_1_*T* + log((1 − *Z*) exp(−*bS*) + Z(1 + exp(*c*)*S*/max *S*)^−1^)
2	log(*R*) − log(*S*) = *a*_0_ + log((1 − *Z*) exp(−*bS*) + Z(1 + exp(*c*)*S*/max *S*)^−1^)
3	log(*R*) − log(*S*) = *a*_0_ + log(exp(−*bS*))
4	log(*R*) − log(*S*) = *a*_0_ − log(1 + exp(*c*)*S*/max *S*)
5	log(*R*) − log(*S*) = *a*_0_ + log(exp(−*b*(1 − *Z*)*S*))
6	log(*R*) − log(*S*) = *a*_0_ − log(1 + exp(*c*(1 − *Z*))*S*/max *S*)

A model formulation on the logarithmic scale (table 1, model 1) is, however, preferred because after the log transformation, the assumption of a constant-variance normal error distribution appears to be met (see electronic supplementary material). In turn, this allows us to estimate the model by minimizing the nonlinear least-squares objective function. For this purpose, we used the *nls* function provided in program R [35]. Here, a Gauss–Newton algorithm is used to determine the nonlinear least-squares estimates of the model parameters. Note also that the term log(*S*) is not moved to the right side of the equation for the subtle technical reason that the *nls* function does not have an offset option.

The statistical support for this model is compared with that of several alternative candidate models. First, we tested a combined Ricker–Beverton–Holt model including the zooplankton covariate but excluding the sea temperature covariate (table 1, model 2). Second, we fitted each of the traditional Ricker and Beverton–Holt models (table 1, models 3 and 4). Finally, we added two models to the list in order to explore whether a more parsimonious explanation is for food availability to directly affect either of the traditional Ricker or Beverton–Holt models. We hypothesized that low food availability (1—zooplankton) might lead to increased overcompensation (table 1, model 5), or it might lower the carrying capacity of the system (table 1, model 6). Additional models involving for instance a temperature effect within each of the Ricker and Beverton–Holt models might also be formulated, but following the model selection philosophy outlined by Burnham & Anderson [36], we include only a restricted set of *a priori* defined models needed for testing our hypotheses. Model selection was based on the Akaike Information Criterion (AIC; [36]), where the model with the lowest AIC value represents the best compromise between bias (including too few parameters) and lack of precision (including too many parameters). The model with the lowest AIC value will therefore have most support and was used in making inferences about North Sea cod recruitment.

## 3. Results

**Figure 2.**
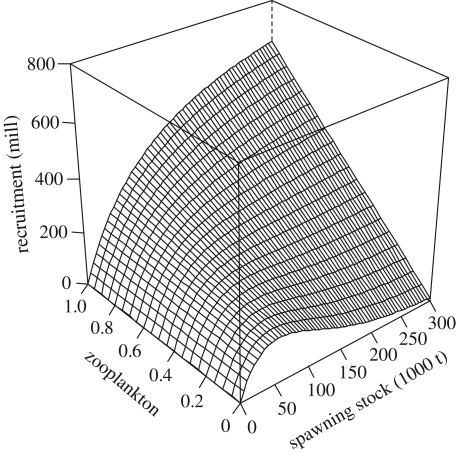
Influence of spawning stock biomass and pelagic food availability (zooplankton) on North Sea cod recruitment at age 1, as predicted from a combined Ricker–Beverton–Holt model (table 1, model 1). Predictions are shown for the average annual SST of 10°C.

**Figure 3.**
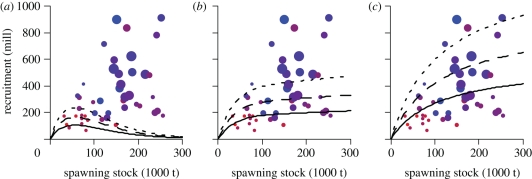
Recruitment of North Sea cod at the historical maximum SST (solid line), average temperature (dashed line) and historical minimum temperature (dotted line) for the corresponding historical (*a*) low food abundance (*b*) average food abundance and (*c*) historical top food abundance as predicted from a combined Ricker–Beverton–Holt model (table 1, model 1). Colour symbols refer to observed combinations of stock and recruitment. The size of the symbols is scaled to reflect annual variation in zooplankton abundance, while the corresponding colour reflects annual SST (red: warm, blue: cold).

The combined Ricker–Beverton–Holt model, where the North Sea cod stock–recruitment relationship is influenced by both zooplankton abundance and sea temperature, had the lowest AIC value and therefore the most support from the available data series (table 2; *a*_0_ = 2.06, s.e. = 0.40, *p* < 0.001; *a*_1_ = 0.18, s.e. = 0.09, *p* = 0.05; *b* = 0.018, s.e. = 0.007, *p* = 0.01; *c* = 0.96, s.e. = 0.58, *p* = 0.10). This model fitted the data reasonably well (Shapiro–Wilk test of normality: *p* = 0.89, see also electronic supplementary material). When the zooplankton index was low, the model indicated a positive relationship between spawning stock biomass and recruitment until the stock reached about 50 000 tonnes; above this level, the model predicted a negative relationship between spawning stock and recruitment (i.e. a Ricker type relationship: figure 2). At intermediate to high zooplankton levels, the model indicated a Beverton–Holt type stock–recruitment relationship, where the recruitment curve levelled out more slowly as zooplankton abundance improved (figure 2). In addition to the zooplankton effect, model selection also supported a negative effect of increasing sea temperature on cod recruitment (table 2 and figure 3). Removing the effect of sea temperature from the model led to an increased AIC value and a corresponding decrease in model support (table 2). The traditional Ricker and Beverton–Holt models received virtually no support when compared with the combined model (table 2). Including a zooplankton effect in each of the traditional Ricker and Beverton–Holt model increased their *r*^2^-value and decreased the corresponding AIC (table 2, models 5–6). Of the two, the Beverton–Holt model containing a zooplankton effect had the most support, indicating that the maximum recruitment would be negatively affected by low food availability (results not shown). The differences in AIC among the four best models were not very large (1–4 units, table 2). Still, the combined Ricker–Beverton–Holt model (with or without a temperature effect) had the most support from the data (table 2, model 1–2).

**Table 2.** Model selection. (The number of parameters to be estimated (*K*), the AIC value (−2log likelihood + 2*K*), the residual sum of squares (RSS) and the proportion of the variance explained (*r*^*2*^) by each of the candidate stock–recruitment models (table 1).)

model	*K*	AIC	RSS	*r*^2^
1	4	62.3	8.43	0.45
2	3	64.6	9.27	0.39
3	2	80.4	13.77	0.10
4	2	80.6	13.81	0.097
5	2	66.6	10.12	0.34
6	2	65.6	9.90	0.35

## 4. Discussion

In this study, we have developed a modelling approach that—when applied to the North Sea cod—demonstrates how the underlying relationship between the population of spawners and recruit fish can be shaped by food availability for fish larvae and sea temperature. We find empirical support for the theoretical prediction [5] stating that the classical Ricker model involving overcompensation [16] may apply when food availability is poor while the classical Beverton–Holt model without overcompensation [15] may apply when food is abundant. We acknowledge that our results, as is generally the case for statistical data-driven models, only are valid within the range of observed values. It follows that future values of environmental conditions (e.g. owing to climate change), spawning stock biomass or recruitment could fall outside this range and that the model then would need to be re-parametrized to remain valid. Future values might also prove helpful in discerning the stock–recruitment relationship at combinations of very low food abundance and very high spawner biomass, and vice versa, where the current data availability was relatively sparse.

Although studies of environmental effects on cod recruitment are numerous, North Sea cod was until very recently probably the only stock for which correlations between time series of planktonic prey and recruitment are established [37]. Thanks to Russian zooplankton data recently being made available such relations have now also being studied for Arcto-Norwegian (Barents Sea) cod [38]. Cushing [39] suggested that successful recruitment of North Sea cod was dependent on a match between the first-feeding larvae and their prey known as the match–mismatch hypothesis. Later, Beaugrand *et al*. [20]. found strong evidence that changes in the plankton community lead to improved recruitment of North Sea cod during the 1960s and 1970s, known as the ‘gadoid outburst’ [39], and that later unfavourable changes in the plankton community intensified the impact of overfishing and caused a marked reduction in recruitment since the mid-1980s. These unfavourable changes involved a reduction in the abundance of the large calanoid copepod *C. finmarchicus*, largely being replaced by its more temperate–water affiliated co-gener, *C. helgolandicus* [40,41]. While nauplii stages of *C. finmarchicus* are the preferred and often dominant prey of larval North Sea cod [31], the less nutritious nauplii of the autumn-spawning *C. helgolandicus* have never been found in the diet records of larval cod [37]. In later years, the distribution of *C. finmarchicus* has shifted northwards and the diet of larval cod in the North Sea tends to be more dominated by smaller copepod species [37]. In the neighbouring Irish Sea, where the abundances of *Calanus* species are low and highly variable, cod still shows a preference for *Calanus*, not only at the onset of external feeding, but also after metamorphosis [42].

The preferred food of early life stages of cod gradually progresses from mainly copepod eggs to copepod and euphausiid nauplii, then to a copepod dominated diet and finally to a progressive replacement of the copepod-based diet by euphausiids and fish larvae [22]. Although the zooplankton index we apply was designed to reflect the potential prey of larval cod, the size-spectrum also includes food suitable for older stages, notably euphasiids and copepodite stages of *Calanus* species. Admittedly, the use of a composite index, covering several species and months, poses challenges when we want to assess effects on the slope of the stock–recruitment curve. For example, one could expect rapid depletion of zooplankton nauplii to lead to over-compensatory effects, while ongoing competition for euphasiids by later stages of young cod would probably be a more purely compensatory process.

Few studies have suggested density-dependent survival of marine fish larvae. The dominating paradigm is that the larvae are too sparsely distributed to influence the abundance of their planktonic prey and thus cause food limitation at high densities. Density-independent environmental controls are thought to account for most of the variability in the early life [43]. However, there is some observational evidence of density dependence occurring at the larval stage (e.g. on bluefin tuna, [44]; walleye pollock, [45]; cod [38]).

Literature giving empirical support for effects of variability in availability of zooplankton prey on larval cod growth is also sparse. This should by no means be taken as evidence that such a connection does not exist, but rather that such investigations are difficult to conduct under both field and culture conditions. A mesocosm (outdoor enclosure system) study by Van der Meeren & Naess [46] showed that cod larvae which lacked sufficient amounts of energetically favourable prey had low-specific growth rates, while the larvae on the other hand realized their high potential for growth when copepod nauplii were abundant. Furthermore, there is convincing support for survival of a cohort (of both cod and other marine fish) being directly related to growth rates during the pre-recruit period [47–49]. A rapid growth rate through the larval and juvenile stages is thought to increase the probability of survival owing to an enhanced ability to feed and avoid predators [12,50]. In this context, our results of zooplankton regulating larval fish growth and survival and ultimately year class strength indeed make sense.

Our results of North Sea cod recruitment being weak during warm periods finds support in earlier work. Evidence suggests that cod stocks towards the northern limit of the species geographical range tend to produce strong year classes during anomalously warm years while southerly stocks are favoured by temperatures below average [51,52]. The North Sea stock is the southernmost of the large stocks of Atlantic cod in the northeast Atlantic. Its recruitment would thus be expected to be negatively correlated with temperature [52–54]. Furthermore, a critical review of environment–recruitment relations points to one generalization that stands out: ‘correlations for populations at the limit of a species' geographical range have often remained statistically significant when re-examined’ [55]. Temperature could affect recruitment directly through juvenile survival and growth performance [3,21]. Temperature may also limit the available habitat [21,24]. Also, there is good evidence that recent warming of the North Sea is affecting the survival of juvenile cod via its effects on the plankton along a critical thermal boundary [2,4,20]. Our study strongly suggests that if warming continues at the rate projected by the Intergovernmental Panel on Climate Change, it will considerably limit larval cod survival and thereby recruitment, making the reconstitution of the stock difficult. Rising temperatures are expected to lead to an increased rate of decline in North Sea cod abundance as compared with model simulations without climate change [56].

Recruitment modelling is a vital component of the assessment and management of marine fish populations, such as the North Sea Atlantic cod. Nevertheless, our understanding of what regulates the number of young fishes has remained opaque since the birth of fisheries science itself [57]. We certainly do not claim that our study will perfectly clarify the mechanisms underlying stock–recruitment relationships, but it reveals how the underlying recruitment dynamics need not be fixed (i.e. described by one specific stock–recruitment curve), but instead shaped by the prevailing environmental conditions (i.e. food availability and sea temperature). In terms of fishery management and conservation, the most important implication of this finding is probably that there will be interaction effects between harvesting and climate change on population dynamics.

## References

[1] Brander K. M. 2007 Global fish production and climate change. Proc. Natl Acad. Sci. USA 104, 19 709–19 714 10.1073/pnas.0702059104 (doi:10.1073/pnas.0702059104) PMC2148362 18077405

[2] Beaugrand G. Edwards M. Brander K. Luczak C. Ibañez F. 2008 Causes and projections of abrupt climate-driven ecosystem shifts in the North Atlantic. Ecol. Lett. 11, 1157–1168 10.1111/j.1461-0248.2008.01218.x (doi:10.1111/j.1461-0248.2008.01218.x) 18647332

[3] Pörtner H. O. 2001 Climate induced temperature effects on growth performance, fecundity and recruitment in marine fish: developing a hypothesis for cause and effect relationships in Atlantic cod (*Gadus morhua*) and common eelpout (*Zoarces viviparus*). Cont. Shelf Res. 21, 1975–1997 10.1016/S0278-4343(01)00038-3 (doi:10.1016/S0278-4343(01)00038-3)

[4] Beaugrand G. Kirby R. R. 2010 Climate, plankton and cod. Glob. Change Biol. 16, 1268–1280 10.1111/j.1365-2486.2009.02063.x (doi:10.1111/j.1365-2486.2009.02063.x)

[5] Johansen R. 2007 A model for the interaction between gadoid larvae and their nauplii prey. Math. Biosci. 208, 177–192 10.1016/j.mbs.2006.10.005 (doi:10.1016/j.mbs.2006.10.005) 17125803

[6] Hilborn R. Walters C. J. 1992 Quantitative fisheries stock assessment. New York, NY: Chapman & Hall

[7] Myers R. A. Barrowman N. J. 1996 Is fish recruitment related to spawner abundance? Fish. Bull. 94, 707–724

[8] Koslow J. 1992 Fecundity and the stock–recruitment relationship. Can. J. Fish. Aquat. Sci. 49, 210–217 10.1139/f92-025 (doi:10.1139/f92-025)

[9] Iles T. C. 1994 A review of stock recruitment relationships with reference to flatfish populations. Neth. J. Sea Res. 32, 399–420 10.1016/0077-7579(94)90017-5 (doi:10.1016/0077-7579(94)90017-5)

[10] Needle C. L. 2002 Recruitment models: diagnosis and prognosis. Rev. Fish Biol. Fish. 11, 95–111 10.1023/A:1015208017674 (doi:10.1023/A:1015208017674)

[11] Keyl F. Wolff M. 2008 Environmental variability and fisheries: what can models do? Rev. Fish Biol. Fish. 18, 273–299 10.1007/s11160-007-9075-5 (doi:10.1007/s11160-007-9075-5)

[12] Cushing D. H. Horwood J. W. 1994 The growth and death of fish larvae. J. Plankt. Res. 16, 291–300 10.1093/plankt/16.3.291 (doi:10.1093/plankt/16.3.291)

[13] Horwood J. Cushing D. Wyatt T. 2000 Plankton determination of variability and sustainability of fisheries. J. Plankt. Res. 22, 1419–1422 10.1093/plankt/22.7.1419 (doi:10.1093/plankt/22.7.1419)

[14] Horwood J. 1995 Plankton-generated chaos in the modelled dynamics of haddock. Phil. Trans. R. Soc. Lond. B 350, 109–118 10.1098/rstb.1995.0145 (doi:10.1098/rstb.1995.0145)

[15] Beverton R. J. H. Holt S. J. 1957 On the dynamics of exploited fish populations. Fish. Invest. Ser. 2 UK Minist. Agric. Fish 19, 1–533

[16] Ricker W. E. 1954 Stock and recruitment. J. Fish. Res. Bd Can. 11, 559–623

[17] Gallego A. Heath M. 1997 The effect of growth-dependent mortality, external environment and internal dynamics on larval fish otolith growth: an individual-based modelling approach. J. Fish. Biol. 51 (Suppl. A), 121–134

[18] Batten S. D. 2003 CPR sampling: the technical background, materials and methods, consistency and comparability. Prog. Oceanogr. 58, 193–215 10.1016/j.pocean.2003.08.004 (doi:10.1016/j.pocean.2003.08.004)

[19] Cook R. M. Sinclair A. Stefánsson G. 1997 Potential collapse of North Sea cod stocks. Nature 385, 521–522 10.1038/385521a0 (doi:10.1038/385521a0)

[20] Beaugrand G. Brander K. M. Lindley J. A. Souissi S. Reid P. C. 2003 Plankton effect on cod recruitment in the North Sea. Nature 426, 661–664 10.1038/nature02164 (doi:10.1038/nature02164) 14668864

[21] Kell L. T. Pilling G. M. O'Brien C. A. 2005 Implications of climate change for the management of North Sea cod (*Gadus morhua*). ICES J. Mar. Sci. 62, 1483–1491 10.1016/j.icesjms.2005.05.006 (doi:10.1016/j.icesjms.2005.05.006)

[22] Beaugrand G. Kirby R. R. 2010 Spatial changes in the sensitivity of Atlantic cod to climate-driven effects in the plankton. Clim. Res. 41, 15–19 10.3354/cr00838 (doi:10.3354/cr00838)

[23] Björnsson B. Steinarsson A. 2002 The food-unlimited growth rate of Atlantic cod (*Gadus morhua*). Can. J. Fish. Aquat. Sci. 59, 494–502 10.1139/f02-028 (doi:10.1139/f02-028)

[24] Rindorf A. Lewy P. 2006 Warm, windy winters drive cod north and homing of spawners keeps them there. J. Appl. Ecol. 43, 445–453 10.1111/j.1365-2664.2006.01161.x (doi:10.1111/j.1365-2664.2006.01161.x)

[25] Oosthuizen E. Daan N. 1974 Egg fecundity and maturity of North Sea cod, *Gadus morhua*. Neth. J. Sea Res. 8, 378–397 10.1016/0077-7579(74)90006-4 (doi:10.1016/0077-7579(74)90006-4)

[26] Kjesbu O. S. 1989 The spawning activity of cod, *Gadus morhua* L. J. Fish. Biol. 34, 195–206 10.1111/j.1095-8649.1989.tb03302.x (doi:10.1111/j.1095-8649.1989.tb03302.x)

[27] Myers R. A. Mertz G. Bishop C. A. 1993 Cod spawning in relation to physical and biological cycles of the northern North-west Atlantic. Fish. Oceanogr. 2, 154–165 10.1111/j.1365-2419.1993.tb00131.x (doi:10.1111/j.1365-2419.1993.tb00131.x)

[28] Hutchings J. A. Bishop T. D. McGregor-Shaw C. R. 1999 Spawning behaviour of Atlantic cod, *Gadus morhua*: evidence of mate competition and mate choice in a broadcast spawner. Can. J. Fish. Aquat. Sci. 56, 97–104 10.1139/cjfas-56-1-97 (doi:10.1139/cjfas-56-1-97)

[29] Brander K. M. 1994 The location and timing of cod spawning around the British Isles. ICES J. Mar. Sci. 51, 71–89 10.1006/jmsc.1994.1007 (doi:10.1006/jmsc.1994.1007)

[30] Helle K. Pennington M. Bogstad B. Ottersen G. 2002 Early environmental influences on growth of Arcto-Norwegian cod, *Gadus morhua*, from the 0-group to adults. Environ. Biol. Fish. 65, 341–348 10.1023/A:1020581415071 (doi:10.1023/A:1020581415071)

[31] Munk P. 1997 Prey size spectra and prey availability of larval and small juvenile cod. J. Fish Biol. 51 (Suppl. A), 340–351

[32] Shepherd J. 1999 Extended survivors analysis: an improved method for the analysis of catch-at-age data and abundance indices. ICES J. Mar. Sci. 56, 584–591 10.1006/jmsc.1999.0498 (doi:10.1006/jmsc.1999.0498)

[33] Corten A. Lindley J. A. 2003 The use of CPR data in fisheries research. Prog. Oceanogr. 58, 285–300 10.1016/j.pocean.2003.08.008 (doi:10.1016/j.pocean.2003.08.008)

[34] Hill R. C. Adkins L. C. 2001 Colinearity. In A companion to theoretical econometrics (ed. Baltagi B. H.), pp. 256–278 Oxford, UK: Blackwell

[35] R Development Core Team 2009 R: A language and environment for statistical computing. Vienna, Austria: R Foundation for Statistical Computing

[36] Burnham K. P. Anderson D. R. 1998 Model selection and inference: a practical information-theoretic approach. New York, NY: Springer

[37] Heath M. R. Lough R. G. 2007 A synthesis of large-scale patterns in the planktonic prey of larval and juvenile cod (*Gadus morhua*). Fish. Oceanogr. 16, 169–185 10.1111/j.1365-2419.2006.00423.x (doi:10.1111/j.1365-2419.2006.00423.x)

[38] Stige L. C. Ottersen G. Dalpadado P. Chan K.-S. Hjermann D. Ø. Lajus D. L. Yaragina N. A. Stenseth N. Chr. 2010 Direct and indirect climate forcing in a multi-species marine system. Proc. R. Soc. B 277, 3411–3420 10.1098/rspb.2010.0602 (doi:10.1098/rspb.2010.0602) PMC2982221 20538646

[39] Cushing D. H. 1984 The gadoid outburst in the North Sea. J. Cons. Int. Explor. Mer. 41, 159–166

[40] Planque B. Fromentin J.-M. 1996 *Calanus* and environment in the eastern North Atlantic. I. Spatial and temporal patterns of *C. finmarchicus* and *C. helgolandicus*. Mar. Ecol. Prog. Ser. 134, 101–109 10.3354/meps134101 (doi:10.3354/meps134101)

[41] Beare D. J. Batten S. Edwards M. Reid D. G. 2002 Prevalence of boreal Atlantic, temperate Atlantic and neritic zooplankton in the North Sea between 1958 and 1998 in relation to temperature, salinity, stratification intensity and Atlantic inflow. J. Sea Res. 48, 29–49 10.1016/S1385-1101(02)00131-4 (doi:10.1016/S1385-1101(02)00131-4)

[42] Rowlands W. L. Dickey-Collas M. Geffen A. J. Nash R. D. M. 2008 Diet overlap and prey selection through metamorphosis in Irish Sea cod (*Gadus morhua*), haddock (*Melanogrammus aeglefinus*), and whiting (*Merlangius merlangus*). Can. J. Fish. Aquat. Sci. 65, 1297–1306 10.1139/F08-041 (doi:10.1139/F08-041)

[43] Houde E. D. 2008 Emerging from Hjort's shadow. J. Northw. Atl. Fish. Sci. 41, 53–70 10.2960/J.v41.m634 (doi:10.2960/J.v41.m634)

[44] Jenkins G. P. Young J. W. Davis T. L. O. 1991 Density dependence of larval growth of a marine fish, the southern bluefin tuna, *Thunnus maccoyii*. Can. J. Fish. Aquat. Sci 48, 1358–1363 10.1139/f91-162 (doi:10.1139/f91-162)

[45] Duffy-Anderson J. T. Bailey K. M. Ciannelli L. 2002 Consequences of a superabundance of larval walleye pollock *Theragra chalcogramma* in the Gulf of Alaska in 1981. Mar. Ecol. Prog. Ser. 243, 179–190 10.3354/meps243179 (doi:10.3354/meps243179)

[46] Van der Meeren T. Naess T. 1993 How does cod (*Gadus morhua*) cope with variability in feeding conditions during early larval stages? Mar. Biol. 116, 637–647

[47] Anderson J. T. 1988 A review of size dependent survival during prerecruit stages of fishes in relation to recruitment. J. Northw. Atl. Sci. 8, 55–66

[48] Pepin P. 1993 An appraisal of the size-dependent mortality hypothesis for larval fish: comparison of a multispecies study with an empirical review. Can. J. Fish. Aquat. Sci. 50, 2166–2174 10.1139/f93-242 (doi:10.1139/f93-242)

[49] Islam M. S. Ueno M. Yamashita Y. 2010 Growth-dependent survival mechanisms during the early life of a temperate seabass (*Lateolabrax japonicus*): field test of the ‘growth-mortality’ hypothesis. Fish. Oceanogr. 19, 230–242 10.1111/j.1365-2419.2010.00539.x (doi:10.1111/j.1365-2419.2010.00539.x)

[50] Rice J. A. Miller T. J. Rose K. A. Crowder L. B. Marschall E. A. Trebitz A. S. DeAngelis D. L. 1993 Growth rate variation and larval survival: inferences from an individual-based size-dependent predation model. Can. J. Fish. Aquat. Sci. 50, 133–142 10.1139/f93-015 (doi:10.1139/f93-015)

[51] Templeman W. 1972 Year-class success in some North Atlantic stocks of cod and haddock. ICN Spec. Publ. 8, 223–241

[52] Planque B. Fredou T. 1999 Temperature and the recruitment of Atlantic cod (*Gadus morhua*). Can. J. Fish. Aquat. Sci. 56, 2069–2077 10.1139/cjfas-56-11-2069 (doi:10.1139/cjfas-56-11-2069)

[53] Dickson R. R. Pope J. G. Holden M. J. 1973 Environmental influence on the survival of North Sea cod. In The early life history of fish (ed. Blaxter J. H. S.), pp. 69–80 Heidelberg, Germany: Springer

[54] O'Brien C. M. Fox C. Planque B. Casey J. 2000 Climate variability and North Sea cod. Nature 404, 142 10.1038/35004654 (doi:10.1038/35004654) 10724155

[55] Myers R. A. 1998 When do environment-recruitment correlations work? Rev. Fish Biol. Fish. 8, 1–21

[56] Clark R. A. Fox C. J. Viner D. Livermore M. 2003 North Sea cod and climate change: modeling the effects of temperature on population dynamics. Glob. Change Biol. 9, 1669–1680 10.1046/j.1529-8817.2003.00685.x (doi:10.1046/j.1529-8817.2003.00685.x)

[57] Hjort J. 1914 Fluctuations in the great fisheries of northern Europe viewed in the light of biological research. Rapp. P. Reun. Cons. Int. Explor. Mer. 20, 1–228

